# P-2131. Impact of Cytomegalovirus Clearance on Outcomes in Hematopoietic Cell Transplant Recipients with Refractory, Resistance or Intolerance to Treatments: Retrospective Study in Europe, Canada and Israel

**DOI:** 10.1093/ofid/ofaf695.2295

**Published:** 2026-01-11

**Authors:** Johan A Maertens, Shariq Haider, Matthew Cheng, Avichai Shimoni, Ilaria Albieri, François Gavini, Tien Bo, Irmgard Andresen

**Affiliations:** UZ Leuven, Leuven, Vlaams-Brabant, Belgium; Juravinski Hospital and Cancer Center, Hamilton, Ontario, Canada; McGill University Health Centre, Montreal, Quebec, Canada; Chaim Sheba Medical Center, Ramat Gan, Tel Aviv, Israel; Takeda Pharmaceuticals International AG, Zürich, Zurich, Switzerland; Takeda Pharmaceuticals International AG, Zürich, Zurich, Switzerland; Takeda Development Center Americas, Inc., Lexington, MA; Takeda Pharmaceuticals International AG, Zürich, Zurich, Switzerland

## Abstract

**Background:**

Cytomegalovirus (CMV) infection is a risk factor for mortality in hematopoietic cell transplant (HCT) recipients. This real-world analysis describes the impact of viremia clearance on outcomes in HCT recipients with refractory/resistant (RR) CMV infection or intolerance (I) to anti-CMV agents.

**Figure:**
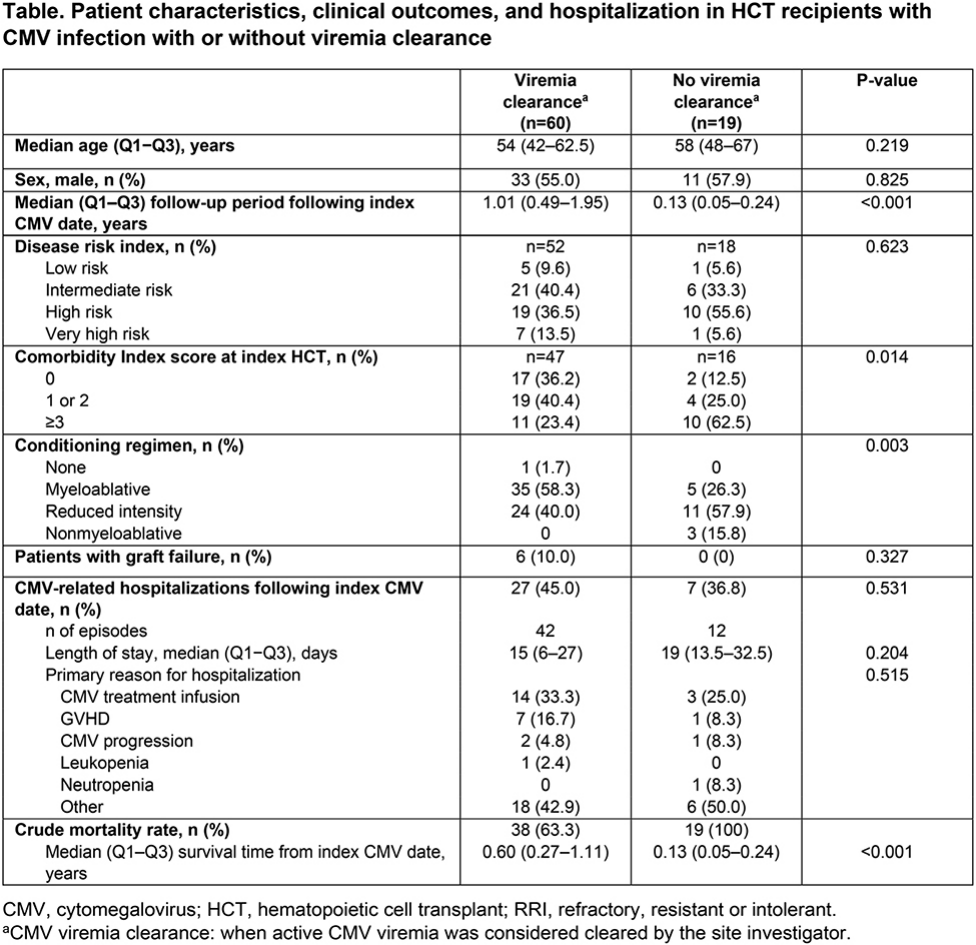

**Figure:**
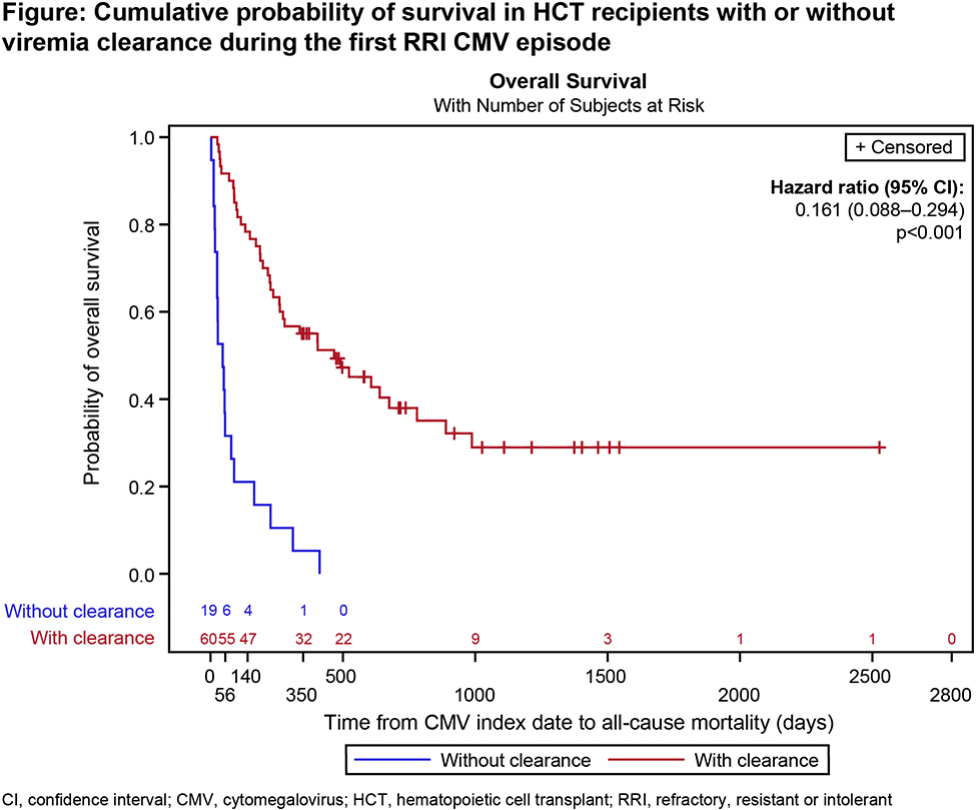

**Methods:**

This multicenter, retrospective, medical chart review included data from adult HCT recipients with RRI CMV in 10 transplant centers in Austria, Belgium, Greece, Poland, Serbia, Canada and Israel. Patient follow-up was ≥ 12 months from the index episode or until death. Patients with vs without viremia clearance during the first RRI CMV episode after transplant (index episode) were compared using Chi-Square or Fisher's Exact tests for categorical variables, independent t-test or Mann-Whitney U tests for quantitative variables, and Cox regression analysis for mortality. All tests were two-sided (significance level, 5%).

**Results:**

Among 79 patients with a first RRI CMV episode, 60 (76%) achieved viremia clearance and 19 (24%) did not. Patient characteristics and outcomes are shown in the Table. Patients with no viremia clearance tended to have higher comorbidity index scores and receive less intensive conditioning regimens than those with viremia clearance. Intermediate/high viral load levels (≥9100 IU/mL) were detected before/at treatment start in 49% vs 31% of patients with vs without viremia clearance. In patients with vs without viremia clearance, the index CMV episode was classified as RR in 68% vs 89% and I in 32% vs 11% of patients. Graft failure occurred in 10% vs 0% and CMV-related hospitalizations in 45% vs 37% of patients with or without viremia clearance, respectively (Table). All patients without viremia clearance died vs 63% with viremia clearance, and survival time from CMV index date was significantly longer with viremia clearance (Table and Figure).

**Conclusion:**

These results highlight the poor prognosis of HCT recipients with RRI CMV infection. In this difficult-to-treat population, the mortality rate was higher in patients without viremia clearance than in patients with viremia clearance, reinforcing the vital role of viremia clearance. The data should be interpreted with caution due to low patient numbers under consideration.

**Disclosures:**

Johan A. Maertens, MD PhD, Amgen: Consulting fees and non-financial support|Astellas Pharma: Honoraria|Astellas Pharma: Consulting fees and non-financial support|Basilea: Consulting fees and non-financial support|Bio-Rad: Grant/Research Support|Bio-Rad: Consulting fees and non-financial support|Cidara: Consulting fees and non-financial support|F2G: Honoraria|F2G: Consulting fees and non-financial support|Gilead Sciences: Grant/Research Support|Gilead Sciences: Honoraria|Gilead Sciences: Consulting fees and non-financial support|Merck: Grant/Research Support|Merck: Consulting fees and non-financial support|Merck Sharpe & Dohme: Honoraria|Mundipharma: Honoraria|Pfizer: Grant/Research Support|Pfizer: Honoraria|Pfizer: Consulting fees and non-financial support|Schering-Plough: Consulting fees and non-financial support|Scynexis: Consulting fees and non-financial support|Shire/Takeda: Consulting fees Shariq Haider, MD, FRCPC, FACP, CCST, Merck: Advisor/Consultant|Takeda: Advisor/Consultant|Takeda: Clinical Trials Matthew Cheng, MD, Amplyx Pharmaceuticals: Grant/Research Support|AstraZeneca: Honoraria|Canadian Institutes of Health Research: Grant/Research Support|Cidara Therapeutics: Grant/Research Support|Fonds de Recherche du Québec – Santé: Grant/Research Support|GEn1E Lifesciences: Advisor/Consultant|GEn1E Lifesciences: Personal fees|Kanvas Biosciences: Ownership Interest|Merck: Honoraria|Nomic Bio: Advisor/Consultant|Nomic Bio: Personal fees|Pfizer: Honoraria|Scynexis: Grant/Research Support|Takeda: Honoraria Ilaria Albieri, PhD, Takeda: Employee|Takeda: Stocks/Bonds (Public Company) François Gavini, MSc, Takeda: Employee|Takeda: Stocks/Bonds (Public Company) Tien Bo, PharmD, Takeda: Employee|Takeda: Stocks/Bonds (Public Company) Irmgard Andresen, MD, Takeda: Employee|Takeda: Stocks/Bonds (Public Company)

